# Nurses' experiences of transitions of older patients from hospitals to community care. A nation‐wide survey in Norway’

**DOI:** 10.1002/hsr2.174

**Published:** 2020-07-16

**Authors:** Heidi Gautun, Christopher Bratt, Jenny Billings

**Affiliations:** ^1^ Norwegian Social Research (NOVA) Oslo Metropolitan University Oslo Norway; ^2^ School of Psychology – Keynes College University of Kent Canterbury UK; ^3^ Department of Psychology Inland School of Business and Social Sciences, Inland Norway University of Applied Sciences – Lillehammer Campus Lillehammer Norway; ^4^ Integrated Care Research Unit, Centre for Health Service Studies University of Kent Canterbury UK

**Keywords:** community care, hospitals, integrated care, older patients, quality of transition

## Abstract

Moving older patients from hospitals to community services is a critical phase of integrated care. Yet there has been little large‐scale research on the quality of these transitions. We investigated how Norwegian nurses working in community care services (N = 4312) and at in‐patient wards at hospitals (N = 2421) experienced the quality of transitions of older patients from hospitals to community care. We tested hypotheses derived from qualitative research and consistent with predictions, we found that compared to hospital nurses, the nurses working in community care experienced lower quality of patient transitions and were less satisfied with information exchange on patients' condition and needs. Further, when comparing groups of community nurses, we confirmed the hypothesis that nurses in home nursing were more dissatisfied with the quality of transitions and information exchange than nurses in nursing homes. We conclude that hospital nurses should have more face‐to‐face or telephone contact with community nurses, and specifically with home nurses. Further, we suggest that means are implemented to promote a mutual understanding of the older patients' pathway from one service to the other, and to improve co‐ordination across the services.

## Graphical abstract

The current paper uses extensive data sets to investigate how nurses working within hospital and community care services experienced the quality of older patients` transitions from hospital to community care. Nurses working in community care reported lower quality than did hospital nurses. Nurses in home nursing more than in nursing home were dissatisfied with the quality of patient transitions.

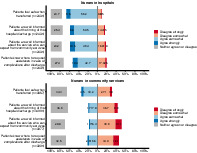

The burden of disease is shifting away from older people suffering from acute illness towards those with long‐term conditions and multiple co‐morbidities^1^, ^2^, ^3^ with increasing numbers of older people needing both treatment in hospital and care in the community.^4^, ^5^ Policy makers are searching for means to improve collaboration and coordination between hospitals and community services.^6^, ^7^, ^8^

Many qualitative studies have looked into patient transitions from hospitals to community services, but large‐scale studies with quantitative data are missing. In the present research, we address this shortcoming in research on integrated care.

## 1 EARLIER RESEARCH ON INTEGRATED CARE FOR OLDER PATIENTS

Many integrated care initiatives have focused on transitions of care between acute hospital and community services.^9^ However, despite international concern for ensuring safe and high‐quality transitions for an increasing number of older people, a scoping review shows that health personnel report significant collaboration problems around patient discharge.^10^ These problems include incomplete exchange of information between healthcare providers and greater challenges in managing and coordinating care delivery to ensure optimal outcomes.^11^, ^12^ Older people in need of care still experience poor quality transitions from hospital to community care, increasing the risk for needs at home not being met and subsequent early readmission to hospital, or unwanted permanent placement in residential care.^13^, ^14^

While there is evidence from qualitative research of transitional difficulties facing professionals who provide care, tests with large datasets using suitable statistical methods is needed to generate evidence‐based recommendations for practice. Using large‐scale data collected in the Norwegian research project “A cross‐sectoral approach to high quality health care transitions for older people, 2016‐2021” (CROSSCARE‐OLD), the aim of this paper is to investigate how Norwegian nurses working within hospital and community care services experience the quality of transitions of older people from acute hospital to community care, and to suggest measures to improve these transitions.

## 2 THE NORWEGIAN EXAMPLE

Alongside other countries, Norway employs integrated care to handle the increasing number of older people in need of both specialist treatment and care in the community.^15^ The Norwegian welfare state is responsible for providing health care and social care services to the entire population, and almost all Norwegian services are public. Therefore, specialist health care, including hospitals, is the responsibility of the central government. Primary care is decentralized to the municipalities, with each municipality obliged to fund and provide primary health as well as long‐term care to its inhabitants.^13^ To improve collaboration and exchange of information between services, Norwegian health authorities have introduced written agreements between administrations at hospitals and municipalities as well and electronic messages in patients' electronic patient records.^16^

Compared with most other European countries, Norway, and the other Nordic countries offer more home‐care services to older people,^8^ and have more residential long‐term facilities for older people. In 2017, 32% of the Norwegian people 80 years or older received home‐care services,^17^ and 13% of the population 80 years or older had a long‐term stay in a nursing home.^18^

## 3 INTEGRATED CARE

The quality of patient transitions from one service to another depends on the services being able to maintain integrated care. There are several definitions of integrated care, we use Kodner & Kyriacou's^7^ definition as it being a*“… set of techniques and organisational models designed to create connectivity, alignment and co‐ordination within and between the cure and care sectors at the funding, administrative and/or provider levels…. for patients with complex problems.”* Their definition reflects the complexity of implementing integrated care‐especially for older people. It also indicates the many transition points that initiatives must address for successful co‐ordination.

When describing how patient transitions are handled between hospitals and community services, we use the term “transition.” Transition refers to the process of change from one form, state, style, or place to another.^19^

### 3.1 Care transition and the coordination of care between professional groups

Care transition is understood as the continuity of health care when the patient is transferred across different health care levels.^20^ Care co‐ordination is intrinsic to safe care transition and is described as navigating people through the health system to prevent unnecessary interruptions in the way care is delivered.^21^ Interventions to improve co‐ordination have become well established across developed countries (ie, ^21^, ^22^, ^23^). But despite policies driving the integrated care agenda, the literature reports continuing problems with transitions of care from hospital to home‐based care where services still struggle specifically with the co‐ordination of care between professional groups (Lloyd & Waits, 2006; Manderson et al., 2011).^12^ Health and social care services offered to patients are still fragmented, lacking continuity, and have inadequate information exchange. Although it is well known that vulnerable populations such as older people are the most in need of better care co‐ordination, they tend to be the least likely to receive co‐ordinated services.^24^, ^25^ Their needs leave them exposed to medical errors, incomplete or inaccurate information, preventable and unnecessary hospital readmission, and even unnecessary death.^24^, ^26^, ^27^ Moreover, older people can suffer from poor discharge routines as care services are delayed, or fail to be delivered at all.^28^, ^29^ Qualitative studies with interviews of geriatric patients and their relatives have uncovered a lack of user participation, user satisfaction and vague responsibilities among staff during care transition, limiting the continuity of care^24^, ^30^, ^31^, ^32^, ^33^(Kvær, Debesay, Bye, Langaas & Bergland, 2019).

There is a lack of large‐scale research investigating how nurses working in hospitals and community care services experience older patients’ transitions from hospital to community care. Qualitative research, however, has been valuable in shedding light on a number of difficulties that persist in limiting the ability of professionals to provide optimal integrated care. This includes a lack of standardized processes and poor multidisciplinary communication across settings, leading to chaotic, unsystematic transitions, poor patient outcomes, and feelings of futility and dissatisfaction among providers.^34^, ^35^ Some research suggests that community nurses more than hospital nurses experience insufficient contact and exchange of information during the discharge process^36^ and the hasty pace at which information is provided may weaken the ability of community nurses to follow up older patients after they have been discharged from hospitals.^37^ A Norwegian survey found a majority of community nurses reporting that older patients were discharged too early from acute hospitals (Author, 2016). This study also suggested that insufficient resources and inadequate information from the hospitals contributed to frequent readmissions soon after hospital discharge. Specifically, more home nurses than nurses working in nursing homes reported a high number of readmissions shortly after discharge.^6^

### 3.2 The need for precise thermology and measurements

One limitation in previous research on the co‐ordination of care is the lack of a consistent terminology. The definition of co‐ordination becomes entangled with terms such as continuity of care, integration, patient‐centered care and case management, all of which also vary in their meaning.^38^ Nevertheless, Uijena et al^38^ identified three core aspects which repeatedly were referred to in the literature as important for the patient: the personal relationships between the patient and the care provider, communication between health and care providers, and cooperation between the providers. Consequently, there is some agreement that co‐ordination of care refers to a person‐centered, assessment‐based, interdisciplinary approach to integrating health care and social support, offered in a cost‐effective way and adapted to the specific needs of individuals and their informal carers. Research shows that a good quality transition, from the older patient’s perspective, implies that the patients are experiencing the transition as safe, that patients are well prepared for leaving the hospitals, and that they receive information about the local services they will receive, as well as information on where to turn if unforeseen events should arise after hospital discharge.^39^ In the present research, we investigate these aspects further as experienced by nurses involved in older peoples' transition from hospitals to community services.

Given the challenges, there is a clear need for further research to acquire knowledge about procedures within organizations, agreements between health and care services, and how employees within health and care services experience co‐ordination when collaborating across health care settings. Specifically, large‐scale quantitative data are now required, and there is a need to balance experiences among community nurses with the comparative experiences of hospital‐based nurses to understand more fully the discrepancies.

### 3.3 Hypotheses

Based on findings from the research literature, we developed the following hypotheses on nurses' experiences:

1. Community nurses report lower quality of transitions than nurses in hospitals do
2. Nurses working in the communities are less satisfied with the information exchange than nurses in hospitals
3. Community nurses more than hospital nurses report insufficient contact with the nurses working in the collaborating services.
4. Nurses in home nursing more than nurses at nursing homes will report dissatisfaction with the quality of transition and information exchange, and also express stronger requests for improved contact with nurses at hospitals.

## 4 METHODS

### 4.1 Data collection and sample

Data were collected in 2017 among Norwegian nurses with two nationwide web‐based surveys, one among nurses working at in‐patient wards in acute hospitals, another among nurses in home nursing and nursing homes in the community. We included only nurses involved in the transfer of patients 65 years or older., and we excluded nurses in the administration of hospitals or municipalities, as well as hospital nurses working in psychiatric care or wards with no in‐patients. We aimed at having a large, national sample rather than a limited sample from a few institutions. However, no national register was available to identify nurses fitting our selection criteria. Therefore, to achieve such a large, national sample, we employed email lists of nurses who were members of the Norwegian Nurses Organisation (NNO), where most Norwegian nurses are organized.

We sent emails that included a link to the online questionnaire to all members of the NNO registered as working in acute hospitals (29 316 nurses) and members registered as working in the municipalities (20 714 nurses). The emails included a recommendation by the NNO to participate in the survey. The two surveys received ethical approval from the Norwegian Social Science Data Services (2017), serving as the Norwegian Data Inspectorate's partner for implementation of the statuary data privacy requirements in the research community (project numbers: 52722 and 53155).

The data collection method prevented the computation of response rates, but provided a data set much more representative for Norwegian nurses caring for older people than other data collection methods would have. Since the email registers in the NNO are not continuously updated, not all nurses receiving the emails were still working in hospitals, nursing homes, or home nursing—some had changed their jobs, gone back to education, were retired, or had long‐term sick leave. Also, our inclusion criteria for the survey excluded many nurses at hospitals and in community services.

The emails requested the nurses outside the target groups not to complete the questionnaire. To further ensure that only nurses fitting the inclusion criteria were included in the analyses, the questionnaire asked the nurses for their current workplace and whether they had been involved in the transition of older patients from hospital to community services. The participating nurses were guaranteed anonymity and answering the questionnaire was considered as informed consent. We sent three reminders—1, 2, and 3 weeks after the original invitation to participate in the survey.

In total, 2431 nurses at in‐patient wards in acute hospitals (94% female) and 4312 nurses in nursing homes and home nursing (94% female) responded to the surveys. Most of Norway's 428 municipalities (88%) were included. Participating hospital nurses worked at different types of acute hospital in‐patient wards and they worked at wards of different sizes. Community nurses worked in municipalities of various sizes, from large cities to municipalities with less than 500 inhabitants. Approximately half of the community nurses (49%) were employed in nursing homes, 45% worked in home care services. We dropped from analyses the remaining 5% who were employed both in nursing homes and home nursing. Seventy three percent of the nurses provided complete data. We used estimation techniques that allowed statistical analyses to include even cases with partially missing data, using full information methods.^40^ The Supporting Information has further details on the samples.

### 4.2 Measurements and analysis

Questionnaire items built on earlier research on discharge of hospital patients.^6^, ^39^, ^41^ To further validate items, we tested them in a pilot survey using random samples of 41 nurses in the community services and 20 nurses in hospitals.

We assessed the quality of transition with four items, the information provided from hospitals to receiving services was assessed with eight items (see below and the Supplemental Materials for details on the items). We used factor analysis to have valid measurements of the quality of transition and information exchange. By using factor analysis, we estimated these dependent variables as latent, cancelling out measurement errors associated with single items (eg, Ref. 42). Confirmatory factor analysis (CFA) corroborated that the four items on the *quality of patient transition* loaded on a single factor—the overall quality of patient transfer. We used exploratory factor analysis (EFA) to analyze the eight items assessing *information exchange from hospitals to community services*. EFA concluded with two factors: information on patients' condition and information on patients' needs (see the Supporting Information for details). We then added predictors of the perceived quality of patient transfer and information exchange, using structural equation modelling, SEM (eg, Ref. 42). All analyses were conducted with Mplus 8.2.^43^

CFA and SEM provide testable models in the sense that in incorrect model may fail by not explaining the data (see for instance Ref. 42). Tests of model fit used the *χ* ^2^, the Comparative Fit Index (CFI), the Root Mean Square Error of Approximation (RMSEA) and its 90% confidence interval, and the Standardized Root Mean Square Residual (SRMR). We used common cut‐off values (eg, Ref. 44) for fit indices (CFI > 0.95; RMSEA preferably below 0.05 and not higher than 0.08; SRMR < 0.08). Consistent with common practice, we did not emphasize the *χ* ^2^, given the large size of the sample. However, to ensure that our findings were robust, we added tests of modified models that achieve perfect fit (*χ* ^2^ based *P* < .05) or close to perfect fit, controlling that these exploratory modified models confirmed the associations between variables indicated by the original models (see the Supporting Information for details). In addition to effect sizes, we include two‐sided *P*‐values. Tables in the Supporting Information show 95% confidence intervals.

## 5 RESULTS

### 5.1 The quality of patient transitions from hospitals to community services

**FIGURE 1:**
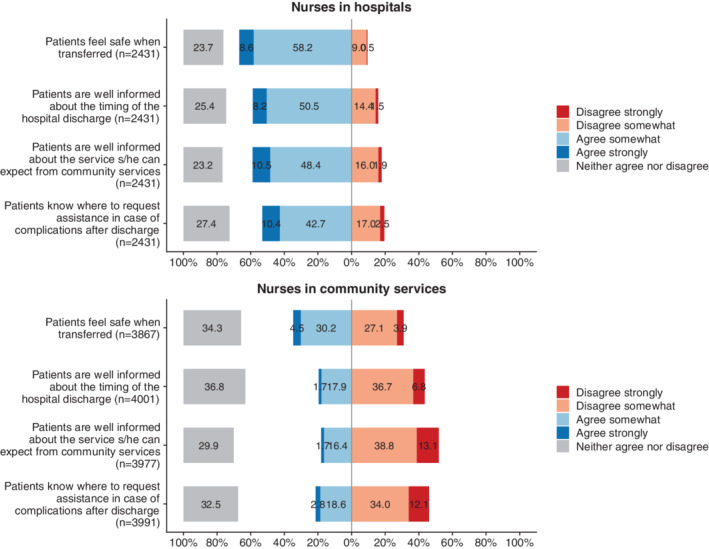
Frequency plots for responses to items on transfer quality, separate results for nurses in in hospitals and nurses in services

As predicted, nurses in community services reported lower quality of patient transitions than did nurses in hospitals (see Figure 1 for frequencies and Table 1 for detailed statistical analyses). Community nurses and hospital nurses expressed very different views—a regression weight of −0.72 for working in a nursing home on the reported quality of patient transitions implied a reduced score of 0.72 on the five‐point scale for the quality of patient transitions.

**TABLE 1:** Nurses' experiences of the quality of transition, information on patients' condition, and patients' needs, dependent on where nurses worked

	Quality of transition		Information on condition		Information on needs	
	Estimate	*P*	Estimate	*P*	Estimate	*P*
*Regression weights*						
Nursing home	−0.72	<.001	−1.28	<.001	−0.86	<.001
Home nursing	−0.96	<.001	−1.49	<.001	−0.98	<.001
Nursing home → Function			−0.67	<.001		
Home nursing → Function			−0.60	<.001		
*Model fit*						
R‐squared	0.28		0.37		0.39	
Chi‐square	367.74		82.08		244.23	
df	8.00		6.00		6.00	
*P*	.00		.00		.00	
CFI	0.98		1.00		0.99	
RMSEA	0.08		0.04		0.08	

*Note:* Hospital nurses were the reference group, with which nurses at nursing homes and at home nursing were compared. Achieving model fit for the SEM analysis of information on patients’ condition required separate paths to the item on patients functioning (see also Figure 1). Further details are available in the Supporting Information.

Abbreviations: CFI, Comparative Fit Index; RMSEA, Root Mean Square Error of Approximation.

The analysis also confirmed that nurses in home nursing had more unfavorable experiences of transition quality than did nurses in nursing homes: Regression weights were consistently stronger (with a negative weight) for home nursing that for nursing homes (Table 1), with nonoverlapping confidence intervals (see the Supporting Information for details).

### 5.2 Information on patients' condition and needs

**FIGURE 2:**
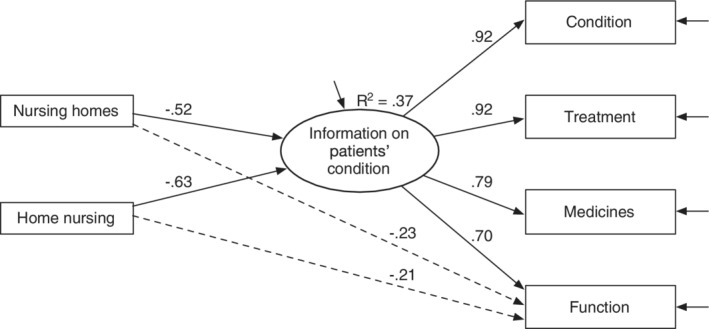
Path diagram information exchange on patients' condition, standardized estimates*. Note:* The model required two exploratory developed paths, indicated by broken lines. See Table 1 and the Supporting Information for details

Even opinions on information exchange on patients' condition and needs differed between hospital and community nurses. Again, community nurses expressed more negative views (see Table 1, see also Figure S1 in the supplemental materials for an illustration). Discrepancies were particularly large for the information on *patients' condition*, as shown in Table 1 and Figure 2. This model required direct paths from nurses' workplace to information on patients' functioning (see the two broken paths in Figure 2), indicating that information on patients' functioning tended to be particularly unsatisfying for community nurses.

### 5.3 Contact between hospitals and community services

Community nurses expressed clearly more negative views on the quality of the transitions, and on the information provided for community services. We note that these differences could not be explained by nurses' age or education level, see the Supporting Information, Table S8, for details.

One possible explanation of unsatisfying quality of transitions could be that the services had little direct contact. Nurses in both community services reported a stronger request for increased contact across services during patient transfer than did nurses in hospitals (the regression weights were 0.35 [95% CI = 0.57, 0.70] for nursing homes and 0.64 [0.29, 0.42] for home nursing). Furthermore, as indicated by the two regression weights, nurses in home nursing more than in nursing homes were unsatisfied with the existing contact: The regression weight for home nursing was nearly double the size of the regression weight for nursing homes, with nonoverlapping confidence intervals.

We also tested the request for more contact as a predictor of the reported quality of patients' transition and views on the information provided. As expected, requests for increased contact was associated with more negative reports on the quality of transition and information exchange, both in community services and in hospitals (Table S9 in the Supporting Information gives details).

Actual contact predicted expressed wishes for more contact, with moderate associations, but stronger in nursing homes, *R* ^2^ = 0.04, than home nursing, *R* ^2^ = 0.02 (see Table S9 in the Supporting Information for details). Phone calls prior to patients' transfer and phone calls by the hospital at the day of transfer predicted higher satisfaction with contact. Also, having nurses from hospitals visiting after the transfer appeared to contribute favorably to the experiences among nurses in home nursing.

Actual contact might also explain experienced quality of transition and experiences of information exchange. Table 2 focuses on these associations. As shown in the table, contact was associated with more favorable experiences, with one exception: outgoing calls on the day of transfer. We return to this finding in the Discussion.

**TABLE 2:** Forms of contact between community services and hospitals as predictors of experienced quality of transition, information on patients' condition, and patients' needs

	Nursing homes		Home nursing		Hospitals	
	Estimate	*P*	Estimate	*P*	Estimate	*P*
*Quality of transition*						
Prior phone call	0.04	.054	0.06	.007	0.05	.001
Visited hospital	0.05	.055	0.10	<.001	0.05	.003
Called out	−0.04	.047	−0.03	.177	−0.04	<.001
Call came in	0.04	.018	0.08	<.001	0.03	.045
Visited community	0.06	.022	0.06	.022	−0.02	.418
*R* ^2^	0.02		0.06		0.02	
*Patients' condition*						
Prior phone call	0.05	.049	0.02	.370	0.02	.363
Visited hospital	0.03	.364	0.00	.928	−0.02	.411
Called out	−0.08	.001	−0.06	.014	0.04	.034
Call came in	0.12	<.001	0.05	.032	0.02	.255
Visited community	−0.02	.714	0.07	.041	−0.11	.006
*R* ^2^	0.04		0.01		0.01	
*Patients' needs*						
Prior phone call	0.07	.001	0.07	<.001	0.06	<.001
Visited hospital	0.06	.009	0.06	.011	0.05	.003
Called out	−0.06	.001	−0.04	.025	−0.03	.047
Call came in	0.10	<.001	0.08	<.001	0.01	.514
Visited community	0.12	<.001	0.09	.001	0.03	.261
*R* ^2^	0.08		0.08		0.02	

*Note:* Prior phone call = telephone conversation prior to the transition, Visited hospital = community care visited the hospital prior to the transition, Called out/Call came in = oneself calling the other sector (out) or the other sector calling (came in), Visited community = representative of hospital visited the community care after the transition. These estimated associations for types of contact are directly comparable, as all used five‐point scales. Detailed model fit estimates are available in the Supporting Information.

## 6 DISCUSSION

Despite policies attempting to improve integrated care, the literature has reported continuing problems with transitions of care from hospital to home‐based care. Services still struggle with coordinating care between professional groups. Consistent with our hypotheses, the current analysis of two large, national surveys showed that community nurses reported substantially lower quality of patient transitions than did hospital nurses. Furthermore, community nurses more than hospital nurses reported insufficient information exchange, and they were more dissatisfied with the contact between the services during the discharge of patients. Further, comparing the two groups of community nurses showed that nurses in home nursing more than in nursing homes were dissatisfied with the quality of patient transitions and information exchange between services.

The analyses indicated higher risk for unsatisfying transitions of older patients when services had little direct contact. Furthermore, nurses in community services and in hospitals who requested more contact between the services also expressed more negative views on the quality of transition and information on patients' condition and needs.

### 6.1 Implications

The analyses indicate that written agreements and e‐messages as tools to formalize collaboration between hospital and community healthcare, are not sufficient for ensuring good transitions for older patients. Interventions to promote more telephone and face‐to‐face contact between services discharging and receiving patients are needed. The analyses confirmed a link between contact across services and the quality of transitions as well as information exchange, albeit with moderate associations. The associations were moderate probably because the nurses reported on their general views on contact between services and the overall quality of transitions, not case‐by‐case experiences. Case‐by‐case evaluations would probably show stronger associations.

Nurses in home nursing gave more negative reports compared with nurses in nursing homes. Apparently, the standard patterns of communications established by hospitals may fit better for nursing homes than for home nursing. It is probably easier for hospital nurses to communicate with nursing homes than with home nursing. Nurses in home nurses usually work on their own, visiting patients in their homes. They have few opportunities for sharing information with colleagues, and they have fewer opportunities to communicate with hospitals than nurses in nursing homes have.

Our research underlines the importance of improved information exchange and timely contact between services when older patients are transferred from hospitals to community services. This finding is echoed within the international literature (eg, Manderson et al., 2011)^6^, ^12^, ^16^, ^37^ and is now, through this research, reported on a wider and more generalizable scale. Integrated care interventions specific to discharge planning have long sought to bridge this gap, particularly through the formation of multi‐disciplinary discharge teams^45^ with varying levels of success.^46^ A systematic literature review undertaken by Manderson et al. (2011) focused on system navigation as a strategy to address the transitions between services for older people with chronic disease. The authors provided some evidence that integrated and coordinated care guided by a navigator, using a variety of interventions such as care plans and treatment goals, is beneficial for chronically ill older adults transitioning across care settings. As with many integrated care initiatives, the authors pointed out a need for additional research to assess the effectiveness and cost of different approaches to the health system. Although the success of integrated care initiatives is context dependent, in that what will work in one area may not work in another, these examples indicate a potential way forward for professionals to gain an improved mutual understanding of the older patient's pathway through different sectors.

### 6.2 Strengths and limitations

The present research provides extensive data on nurses' experiences of the quality of transitions of older patients from hospitals to community services. To our knowledge, no earlier research has included similarly extensive data on the quality of transitions of older patients to community services. Another strength of the present research is the use of factor analysis and SEM, providing more reliable findings than analyses of single items or indexes would do.

The sample sizes available in the present research were only possible by using nurses as informants. By allowing nurses to indicate their experiences with the transition of older patients, we involved the professional group with the most detailed knowledge of each patient. An alternative approach could have been to use older patients themselves as informants, specifically patients who recently had been discharged from hospitals and transferred to community services. Such an approach has merits in providing first‐hand information on older patients’ experiences and have be used by several earlier studies. However, using older patients as informants would have given much smaller sample sizes. Moreover, these samples of patients would by design have been biased, because they would have been unable to include many severely ill patients, for instance patients being in advanced stages of dementia. Although the nurses could not give first‐hand reports on the patients’ experiences, they had the advantage of being able to reflect experiences of many older patients transferred from hospitals, not only those patients who would have been able to participate in interviews.

## 7 CONCLUSION

More direct contact between hospital nurses and community nurses is needed, as are interventions that promote a more mutual understanding of the older patients' journeys through the transition with an emphasis on improving care co‐ordination. One means to achieve this aim is to use multi‐disciplinary discharge teams, another can be to develop case management or care navigator roles specific to the discharge process.

## FUNDING INFORMATION

The study is part of the CrossCare‐Old project, a cross‐sectoral approach to high quality health care transitions for older people. This project is funded by the Research Council of Norway (RCN, project no. 256644). The RCN played no role in the design of the study, analysis or interpretation of the data, or in writing the manuscript.

## CONFLICTS OF INTEREST

None conflicts of interest declared.

## AUTHORS CONTRIBUTIONS

Conceptualization: Heidi Gautun, Jenny Billings

Formal Analysis: Christopher Bratt

Funding Acquisition: Heidi Gautun

Methodology: Christopher Bratt

Project Administration: Heidi Gautun

Writing – Original Draft Preparation: Heidi Gautun, Christopher Bratt, Jenny Billings

Writing – Review and Editing: Heidi Gautun, Christopher Bratt, Jenny Billings

All authors have read and approved the final version of the manuscript.

Heidi Gautun had full access to all of the data in this study and takes complete responsibility for the integrity of the data and the accuracy of the data analysis.

## TRANSPARENCY STATEMENT

Heidi Gautun affirms that this manuscript is an honest, accurate, and transparent account of the study being reported; that no important aspects of the study have been omitted; and that any discrepancies from the study as planned (and, if relevant, registered) have been explained.

## DATA AVAILABILITY STATEMENT

The survey data collected for this study are not publicly available because data is required to be stored on a password‐protected file on the secure server at Oslo Metropolitan University to ensure confidentiality. Access to the data was only permitted to the investigators for a designated period of time. After this designated time, a de‐identified dataset, without information on municipality, will be made available for research purposes through the archives at NSD The Norwegian Centre for Research Data.

## Supporting information


**Appendix S1.** Supporting information.
