# Supplementary material for: Nurses' experiences of transitions of older patients from hospitals to community care. A nation‐wide survey in Norway’
Source: Health Sci Rep. 2020 Jul 16;3(3):e174. doi: 10.1002/hsr2.174 (PMC7365956; doi:10.1002/hsr2.174)
Supplement: Supplementary file 1 — Appendix S1. Supporting information. [file HSR2-3-e174-s001.pdf]

Discharging older patients from hospitals – Nurses' experiences of the quality of the transition to community care. Supporting information

Contents

1 Introduction **3**

2 Measurements **3**

3 Descriptive statistics **4**

4 Factor analyses of items on experienced quality of transitions and information exchange **9**

5 Predictors of experienced quality of transitons and information exchange **10**

6 References **13**

7 Appendix: Developing models with perfect fit **14**

List of Tables

S1 Missing data - frequencies and percents 4
S2 Background of participating nurses 5
S3 Workplace of participating nurses 6
S4 Spearman correlations for all items, part 1 7
S5 Spearman correlations for all items, part 2 7
S6 Spearman correlations for all items, part 3 7
S7 Regression weights in analyses of (1) experienced quality of patient transitions, (2) information exchange on patients' condition, and (3) information exchange on patiens' needs. Unstandardised estimates with confidence intervals. 10
S8 Regression weights in analyses of (1) experienced quality of patient transitions, (2) information exchange on patients' condition, and (3) information exchange on patiens' needs. Unstandardised estimates, age and education added. 11
S9 Requesting more contact, dependent on reported contact 11
S10 Forms of contact predicting (1) experienced quality of patient transitions, (2) information exchange on patients' condition, and (3) information exchange on patiens' needs. Separate results for nursing homes, home nursing, and hospitals. 12

List of Figures

S1 Views on information exchange between services during transtion of older patients from hospitals to community services) 8
S2 Factor models of (a) experienced quality of transfer and (b) information exchange (with cross-loadings and correlated residuals in grey) 9
S3 Models with perfect or close to perfect fit, (a) experienced quality of patient transitions, (b) information exchange on patients' condition, and (c) information exchange on patiens' needs. Added parameters (paths) use indicated by broken lines. 15

1 Introduction

This Supporting Information reports on descriptive statistics and details on statistical analyses, summarised in the main manuscript. We also include an Appendix in this Supporting Information, showing results of exploratory modified models to achieve perfect or close-to-perfect model fit.

The document is compiled with R and RStudio, using the `knitr` package (Xie, 2016). Mplus analyses were integrated with R using the `MplusAutomation` package (Hallquist and Wiley, 2018). Tables were developed with `kableExtra` (Xhu, 2019), plots with `ggplot2` (Wickham, 2009).

2 Measurements

Dependent variables in this research were nurses' experiences of the quality of patient transitio and information exchange between services. The quality of patient transition was assessed with four items, asking respondents to indicate to what degree they agreed (on a five-point scale) with the following statements:

- "Patients feel safe when transferred"
- "Patients are well informed about the timing of the hospital discharge"
- "Patients are well informed about the service s/he can expect from community services"
- "Patients know where to request assistance in case of complications after discharge".

The questionnaire included eight items on the information provided by hospitals for community services. The following sentence introduced the eight items: "During discharge of older patients, how common or uncommon is it that sufficient information is given to the community services. The eight items specified the following aspects of information:

- "the patient's medical condition"
- "treatment the patient has received"
- "patient's level of functionality"
- "practical procedures"
- "medication"
- "medical advice"
- "patient's social needs"
- "patients' need for continuing nursing care".

All these items used a five-point ordinal response format (Very uncommon, Uncommon, Sometimes, Common, Very common). In addition, a "Not sure" option was provided, which we coded as missing data.

Contact between hospitals and community services was assessed with items asking

- whether services usually were in telephone contact prior to patient transfers
- whether a representative for the community service visited the hospital prior to the transfer
- whether calls were made between discharging and receiving service the same day as the transfer of older patients (with separate items for community services or hospitals initiating these telephone calls)
- whether a representative of the hospital visited the community service after the patient had been transferred.

Table S1: Missing data - frequencies and percents

Home nursing			Nursing homes			Hospitals		
	Freq	Prct		Freq	Prct		Freq	Prct
Prior telephone call	342	15	Prior telephone call	403	20	Info on social needs	95	4
Education	270	12	Education	316	16	Info on medical advice	94	4
Age	259	11	Age	306	15	Info on procedures	57	2
Pat. felt safe	203	9	Pat. felt safe	242	12	Incoming call	46	2
Incoming call	192	8	Info on condition	222	11	Info on nursing	43	2
Info on condition	185	8	Wants more contact	216	11	Info on function level	31	1
Wants more contact	179	8	Info on function level	205	10	Info on medication	30	1
Info on function level	165	7	Incoming call	203	10	Info on condition	27	1
Info on treatment	163	7	Info on medical advice	199	10	Info on treatment	27	1
Info on nursing	159	7	Info on nursing	198	10	Community visited	27	1
Info on medical advice	158	7	Info on treatment	184	9	Outgoing call	25	1
Hospital visited	157	7	Info on procedures	183	9	Prior telephone call	24	1
Info on medication	155	7	Info on social needs	182	9	Hospital visited	18	1
Pat. well informed	154	7	Hospital visited	182	9	Education	2	0
Info on social needs	154	7	Pat. well informed	181	9	Age	1	0
Info on procedures	150	7	Info on medication	180	9	Pat. felt safe	0	0
Pat. prepared	148	6	Pat. know where to go	179	9	Pat. prepared	0	0
Pat. know where to go	142	6	Community visited	168	8	Pat. well informed	0	0
Outgoing call	111	5	Pat. prepared	163	8	Pat. know where to go	0	0
Community visited	107	5	Outgoing call	159	8	Wants more contact	0	0

Note:
Pat. = patients, Info = Information

All these items used a six-point response format (Have no contact, Very uncommon, Uncommon, Sometimes, Common, Very Common). In addition, a "Not sure" option was provided as a possible answer, which would be coded as missing data. Beyond these items on actual contact, the questionnaire also asked the nurses whether they agreed with the statement "Nurses in community services and nurses at the in-patient ward [in the hospital] should have more contact regarding older patients who are discharged". This item used a five-point Likert response format, indicating strength of (dis)agreement.

3 Descriptive statistics

The samples had few cases with incomplete data, even after we recoded "don't know" answers as missing data (Table S1). Table S2 shows background statistics for participating nurses, Table S3 gives an overview of the nurses' workplace. Table S4 shows correlations between items.

The article includes a figure illustrating differences between hospital nurses and community nurses in their views on the quality of patient transtion. Figure S1 here in the supplemental materials illustrate differences in views on the information exchange between these services.

Table S2: Background of participating nurses

	Hospital	Community
Age		
30 years or younger	40%	19%
31-40 years	26%	28%
41-50 years	17%	26%
51-60 years	13%	21%
61 years or more	4%	5%
Number of years working as a nurse		
0-5 years	41%	23%
6-10 years	20%	19%
11-15 years	13%	18%
16-20 years	9%	16%
21 years or more	17%	24%
Number of years working at their current workplace		
5 years or less	52%	46%
6-10 years	22%	25%
11-15 years	11%	12%
16-20 years	8%	9%
21 years or more	7%	7%
Weekly working hours (incl. extra shifts and second job)		
30 hours or less	23%	18%
31-35 hours	33%	31%
36-38 hours	34%	40%
39 hours or more	10%	11%
Level of education		
None	62%	51%
Less than one year	19%	27%
More than one year	19%	19%
Master	4%	3%

Table S3: Workplace of participating nurses

	Hospitals	Community
Type of community service		
Nursing home		49%
Home nursing		45%
Nursing home and home nursing		5%
Type of department in nursing homes		
Short-term		8%
Long-term		51%
Both short- and long-term		41%
Type of inpatient-ward in hospitals		
Medical	45%	
Surgery	25%	
Combined medical surgery	5%	
Orthopaedic	11%	
Geriatric/older person care	2%	
Other	14%	

Table S4: Spearman correlations for all items, part 1

	1	2	3	4	5	6	7
1. Home nursing							
2. Nursing home		-					
3. Age	0.08***	0.15***	-				
4. Education	0.02	0.07***	0.36***	-			
5. Patients felt safe	-0.29***	-0.04***	-0.02	-0.03*	-		
6. Patients prepared for time of discharge	-0.26***	-0.15***	-0.02	-0.05***	0.58***	-	
7. Patients well informed on community service	-0.32***	-0.13***	-0.03*	-0.06***	0.46***	0.55***	-
8. Patients know where to go if complications	-0.24***	-0.13***	0.03**	-0.03**	0.38***	0.43***	0.49***
9. Information on condition	-0.34***	-0.22***	-0.11***	-0.08***	0.32***	0.36***	0.37***
10. Information on treatment	-0.33***	-0.2***	-0.1***	-0.09***	0.31***	0.34***	0.36***
11. Information on medication	-0.33***	-0.18***	-0.06***	-0.05***	0.29***	0.32***	0.32***
12. Information on function level	-0.38***	-0.29***	-0.12***	-0.09***	0.37***	0.42***	0.44***
13. Information on social needs	-0.3***	-0.15***	0.02*	-0.02	0.31***	0.36***	0.37***
14. Information on procedures	-0.31***	-0.23***	0	-0.04***	0.33***	0.38***	0.4***
15. Information on medical advice	-0.29***	-0.09***	0.1***	0	0.31***	0.36***	0.38***
16. Information on nursing	-0.31***	-0.23***	-0.03*	-0.06***	0.35***	0.39***	0.41***
17. Telephone call prior to discharge	-0.05***	0.21***	0.07***	0.04**	0.02	0	0.03*
18. Incoming call on day of discharge	0.06***	0.34***	0.1***	0.04***	-0.07***	-0.11***	-0.11***
19. Outgoing call on day of discharge	-0.29***	-0.06***	-0.17***	-0.09***	0.12***	0.12***	0.18***
20. Hospital nurse visits community service	0.17***	0.13***	0.13***	0.1***	-0.07***	-0.05***	-0.05***
21. Community nurse visits Hospital	0	-0.09***	0.14***	0.08***	0.08***	0.11***	0.14***
22. Should have more contact	0.21***	0.03*	-0.01	0.03*	-0.26***	-0.29***	-0.28***

Note:
*** p < .001, ** p < .01, * p < .05

Table S5: Spearman correlations for all items, part 2

	8	9	10	11	12	13	14
9. Information on condition	0.31***	-					
10. Information on treatment	0.32***	0.76***	-				
11. Information on medication	0.28***	0.65***	0.64***	-			
12. Information on function level	0.37***	0.67***	0.68***	0.6***	-		
13. Information on social needs	0.34***	0.34***	0.33***	0.3***	0.47***	-	
14. Information on procedures	0.37***	0.53***	0.47***	0.42***	0.54***	0.43***	-
15. Information on medical advice	0.37***	0.45***	0.45***	0.48***	0.49***	0.56***	0.48***
16. Information on nursing	0.38***	0.51***	0.52***	0.45***	0.59***	0.54***	0.53***
17. Telephone call prior to discharge	0.02	-0.04***	-0.04***	-0.04**	-0.05***	0.04**	0.01
18. Incoming call on day of discharge	-0.08***	-0.15***	-0.15***	-0.13***	-0.19***	-0.09***	-0.11***
19. Outgoing call on day of discharge	0.11***	0.19***	0.16***	0.16***	0.22***	0.17***	0.17***
20. Hospital nurse visits community service	-0.04**	-0.16***	-0.14***	-0.13***	-0.16***	0.01	-0.09***
21. Community nurse visits Hospital	0.12***	0.03*	0.02*	0.04***	0.06***	0.13***	0.12***
22. Should have more contact	-0.23***	-0.24***	-0.22***	-0.2***	-0.26***	-0.2***	-0.23***

Note:
*** p < .001, ** p < .01, * p < .05

Table S6: Spearman correlations for all items, part 3

	15	16	17	18	19	20	21
16. Information on nursing	0.56***	-					
17. Telephone call prior to discharge	0.05***	0.01	-				
18. Incoming call on day of discharge	-0.05***	-0.11***	0.38***	-			
19. Outgoing call on day of discharge	0.14***	0.19***	0.29***	0.14***	-		
20. Hospital nurse visits community service	-0.03*	-0.08***	0.16***	0.19***	-0.03**	-	
21. Community nurse visits Hospital	0.11***	0.1***	0.21***	0.08***	0.13***	0.33***	-
22. Should have more contact	-0.22***	-0.22***	-0.03*	0	-0.04***	0.05***	-0.03**

Note:
*** p < .001, ** p < .01, * p < .05

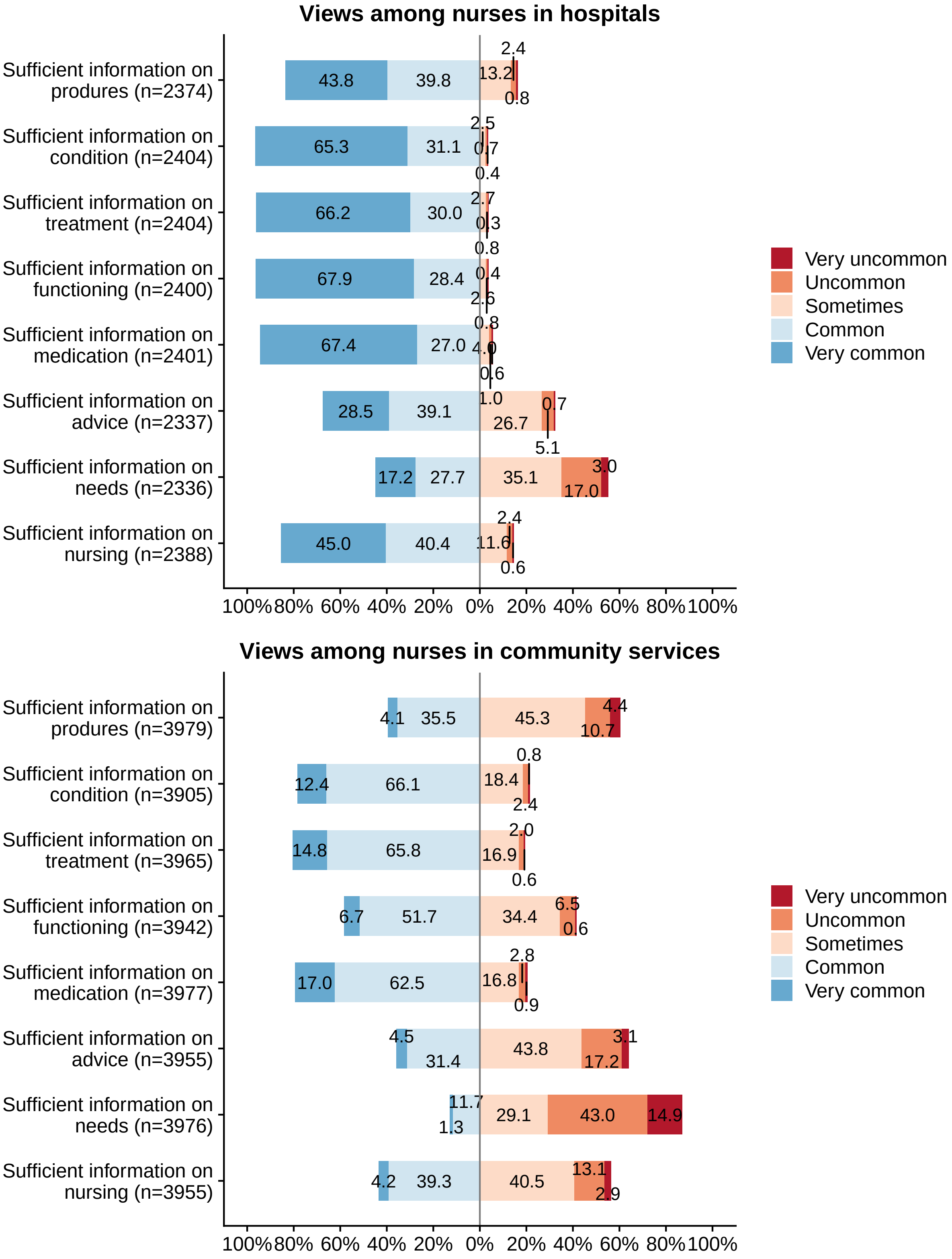

Figure S1: Views on information exchange between services during transtion of older patients from hospitals to community services)

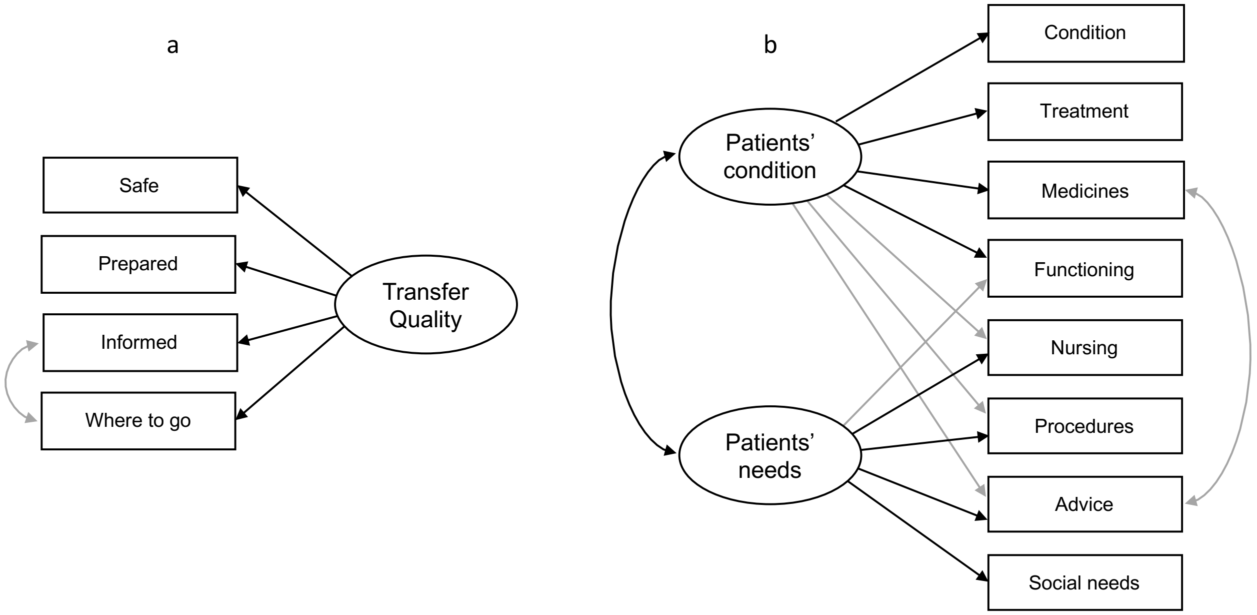

Figure S2: Factor models of (a) experienced quality of transfer and (b) information exchange (with cross-loadings and correlated residuals in grey)

4 Factor analyses of items on experienced quality of transitions and information exchange

We used factor analysis to develop measurement models of experienced quality of transitions and information exchange, with each item defined as ordinal. The *experienced quality of patient transition* was measured with four items (see Figure S1a). This factor model with four items had perfect fit if it included a residual correlation between two closely related items: whether (*a*) the patients were well informed about services s/he would received from local services and (*b*) whether patients knew where to go in case of complications (Chi-square = 2.23; df = 1; $p = 0.14$; CFI = 1; RMSEA = 0.01 [0, 0.04], SRMR = 0.00).

Information exchange across services were assessed with eight items. We used exploratory factor analysis to identify a factor structure for these eight items, which concluded with two dimensions: information exchange on patients' condition and information exchange on patients' needs. A subsequent test with CFA with the two factors gave good fit if we allowed four three crossloadings and a correlation between two residual variables (see Figure S1b): degrees of freedom = 14, Chi-square = 233.02, $p = 0$, CFI = 1.00, RMSEA = 0.05 [0.04, 0.05.] Later analyses with structural equation modelling used only one of the two factors as a dependent variable, meaning that these analyses did not need to add cross-loadings.

Table S7: Regression weights in analyses of (1) experienced quality of patient transitions, (2) information exchange on patients' condition, and (3) information exchange on patiens' needs. Unstandardised estimates with confidence intervals.

	Quality of transition		Information on condtion		Information on needs	
	Estimate	95% CI	Estimate	95% CI	Estimate	95% CI
Regression weights						
Nursing home	-0.72	[-0.77, -0.67]	-1.28	[-1.35, -1.22]	-0.86	[-0.91, -0.80]
Home nursing	-0.96	[-1.01, -0.91]	-1.49	[-1.55, -1.42]	-0.98	[-1.04, -0.93]
Nursing home -> Functioning			-0.67	[-0.73, -0.61]		
Home nursing -> Functioning			-0.60	[-0.66, -0.54]		
R-squared	0.28		0.37		0.39	
Model fit						
Chi-square	367.74		82.08		244.23	
df	8.00		6.00		6.00	
p-value	0.00		0.00		0.00	
CFI	0.98		1.00		0.99	
RMSEA	0.08		0.04		0.08	
90% CI RMSEA, Lower	0.08		0.04		0.07	
90% CI RMSEA, Upper	0.09		0.05		0.09	
SRMR	0.03		0.01		0.02	

5 Predictors of experienced quality of transitons and information exchange

Table S7 is similar to Table 1 in the main manuscript, but Table S7 uses 95% confidence intervals. The next table, Table S8, shows results in models that included nurses' age and education as additional predictors. Neither age nor education had any substantial effect on experienced quality of patient transition; adding these predictors had minor effect on explained variance (R^2), and the effect sizes were close to zero.

We did not use factor analysis for items on reported contact across services since we were interested in associations between specific forms of contact and the experienced quality of transitions or information exchange. The associations were moderate for the single items (see Table S10), as discussed in the article.

Table S8: Regression weights in analyses of (1) experienced quality of patient transitions, (2) information exchange on patients' condition, and (3) information exchange on patiens' needs. Unstandardised estimates, age and education added.

	Quality of transition		Information on condition		Information on needs	
	Estimate	95% CI	Estimate	95% CI	Estimate	95% CI
**Regression weights**						
Nursing home	-0.76	[-0.81, -0.71]	-1.27	[-1.34, -1.20]	-0.96	[-1.01, -0.90]
Home nursing	-0.99	[-1.04, -0.94]	-1.48	[-1.54, -1.41]	-1.08	[-1.14, -1.02]
Age	0.04	[0.03, 0.05]	0.01	[0.00, 0.02]	0.06	[0.05, 0.06]
Education	-0.05	[-0.07, -0.03]	-0.06	[-0.09, -0.03]	-0.04	[-0.06, -0.02]
Nursing home -> Functioning			-0.68	[-0.74, -0.62]		
Home nursing -> Functioning			-0.61	[-0.67, -0.54]		
R-squared	0.30		0.37		0.42	
**Model fit**						
Chi-square	403.87		110.20		314.46	
df	14.00		12.00		12.00	
p-value	0.00		0.00		0.00	
CFI	0.98		1.00		0.98	
RMSEA	0.07		0.04		0.06	
90% CI RMSEA, Lower	0.06		0.03		0.06	
90% CI RMSEA, Upper	0.07		0.04		0.07	
SRMR	0.03		0.01		0.03	

Table S9: Requesting more contact, dependent on reported contact

	Nursing homes		Home nursing		Hospitals	
	Estimate	95% CI	Estimate	95% CI	Estimate	95% CI
Prior phone call	-0.10	[-0.15, -0.04]	-0.05	[-0.10, -0.00]	0.00	[-0.04, 0.03]
Visited hospital	0.02	[-0.05, 0.08]	-0.04	[-0.10, 0.02]	-0.02	[-0.07, 0.02]
Outgoing call	0.13	[0.08, 0.18]	0.10	[0.05, 0.16]	0.04	[0.00, 0.07]
Incoming call	-0.10	[-0.15, -0.05]	-0.08	[-0.13, -0.03]	-0.05	[-0.08, -0.01]
Visited community	-0.07	[-0.15, 0.01]	-0.08	[-0.15, -0.01]	0.13	[0.05, 0.20]
R-square	0.04		0.02		0.01	

Table S10: Forms of contact predicting (1) experienced quality of patient transitions, (2) information exchange on patients' condition, and (3) information exchange on patiens' needs. Separate results for nursing homes, home nursing, and hospitals.

	Nursing homes		Home nursing		Hospitals	
	Estimate	95% CI	Estimate	95% CI	Estimate	95% CI
**Quality of transition**						
Regression weights						
Prior phone call	0.04	[-0.00, 0.08]	0.06	[0.02, 0.10]	0.05	[0.02, 0.08]
Visited hospital	0.05	[-0.00, 0.09]	0.10	[0.06, 0.15]	0.05	[0.02, 0.08]
Outgoing call	-0.04	[-0.07, 0.00]	-0.03	[-0.07, 0.01]	-0.04	[-0.07, -0.02]
Incoming call	0.04	[0.01, 0.08]	0.08	[0.04, 0.12]	0.03	[0.00, 0.06]
Visited community	0.06	[0.01, 0.12]	0.06	[0.01, 0.12]	-0.02	[-0.08, 0.03]
Explained variance						
R-square	0.02		0.06		0.02	
Model fit						
Chi-square	52.64		92.82		85.71	
df	17.00		17.00		17.00	
p-value	0.00		0.00		0.00	
CFI	0.99		0.98		0.98	
RMSEA	0.04		0.05		0.04	
90% CI RMSEA, lower	0.03		0.04		0.03	
90% CI RMSEA, upper	0.05		0.06		0.05	
SRMR	0.02		0.04		0.03	
**Information on patients' condition**						
Regression weights						
Prior phone call	0.05	[0.00, 0.10]	0.02	[-0.03, 0.07]	0.02	[-0.02, 0.06]
Visited hospital	0.03	[-0.04, 0.10]	0.00	[-0.05, 0.06]	-0.02	[-0.07, 0.03]
Outgoing call	-0.08	[-0.12, -0.03]	-0.06	[-0.10, -0.01]	0.04	[0.00, 0.08]
Incoming call	0.12	[0.08, 0.17]	0.05	[0.00, 0.09]	0.02	[-0.02, 0.07]
Visited community	-0.02	[-0.09, 0.06]	0.07	[0.00, 0.14]	-0.11	[-0.18, -0.03]
Explained variance						
R-square	0.04		0.01		0.01	
Model fit						
Chi-square	24.52		34.22		72.88	
df	17.00		17.00		17.00	
p-value	0.11		0.01		0.00	
CFI	1.00		1.00		1.00	
RMSEA	0.02		0.02		0.04	
90% CI RMSEA, lower	0.00		0.01		0.03	
90% CI RMSEA, upper	0.03		0.04		0.05	
SRMR	0.02		0.03		0.01	
**Information on patients' needs**						
Regression weights						
Prior phone call	0.07	[0.03, 0.11]	0.07	[0.04, 0.11]	0.06	[0.02, 0.09]
Visited hospital	0.06	[0.02, 0.11]	0.06	[0.01, 0.10]	0.05	[0.02, 0.08]
Outgoing call	-0.06	[-0.10, -0.02]	-0.04	[-0.08, -0.00]	-0.03	[-0.05, 0.00]
Incoming call	0.10	[0.06, 0.14]	0.08	[0.05, 0.12]	0.01	[-0.02, 0.04]
Visited community	0.12	[0.06, 0.18]	0.09	[0.04, 0.14]	0.03	[-0.02, 0.09]
Explained variance						
R-square	0.08		0.08		0.02	
Model fit						
Chi-square	69.62		41.27		83.40	
df	17.00		17.00		17.00	
p-value	0.00		0.00		0.00	
CFI	0.98		0.99		0.99	
RMSEA	0.05		0.03		0.04	
90% CI RMSEA, lower	0.04		0.02		0.03	
90% CI RMSEA, upper	0.06		0.04		0.05	
SRMR	0.03		0.02		0.03	

Note:
Prior phone call = telephone conversation prior to the transition, Visited hospital = community care visited the hospital prior to the transition, Outgoing/incoming call = oneselves calling the other sector (out) at the day of transition or the other sector calling (in), Visited community = representative of the hospital visted the community care after the transition. CI = confidence interval, CFI = Comparative Fit Index, RMSEA = Root Mean Square Error of Approximation, SRMR = Standardised Root Mean Square Residual.

6 References

Hallquist, M. N., & Wiley, J. F. (2018). Mplusautomation: An r package for facilitating large-scale latent variable analyses in mplus. Structural Equation Modeling: A Multidisciplinary Journal, 1-18. doi: 10.1080/10705511.2017.1402334

Wickham, H. (2009). Ggplot2: Elegant graphics for data analysis. New York: Springer-Verlag.

Xie, Y. (2016). knitr: A General-Purpose Package for Dynamic Report Generation in R. R package version 1.15.1.

Zhu, H. (2019). Package 'kableExtra'. Retrieved from https://cran.r-project.org/web/packages/kableExtra/kableExtra.pdf

7 Appendix: Developing models with perfect fit

Using CFA and SEM had several advantages for the present research. It allowed for the modelling of measurment errors, thereby giving much improved measurements of experienced quality of patients' transition and information exchange. It also allowed for tests of models (testing whether a relatively simple model was able to reproduce the complex data), an crucial aspect of analyses using multi-item measurements.

As common in research using SEM, our models had approximate, but not perfect fit. We decided to add tests where we exploratory modified models to achieve perfect fit (Chi-square-based p-value below 0,05) or close-to-perfect fit. Our aim was to ensure that these modified models did not suggested any different conclusions for the paths (parameters) of interest in the current research.

Figure S2 shows these models with exploratory added paths (developed using modifications indices, an exploraty method available in structural equation modelling). These modifications gave models with very good fit. The modificatins did not result in qualitatively different conclusions for the regression weights of interest in this research (see Figure S2).

Model fit in exploratory modified models:

Experienced quality of transitions dependent on nurses' work place: Chi-square = 13.194, df = 6.000, p = 0.04, CFI = 1.000, RMSEA = 0.014 [0.003, 0.024], SRMR = 0.008).

Information exchange on patients' condition dependent on nurses' work place: Chi-square = 5.940, df = 3.000, p = 0.115, CFI = 1.000, RMSEA_Estimate = 0.012 [0.000, 0.027], SRMR = 0.005.

Information exchange on patients' needs dependent on nurses' work place: Chi-square = 0.591, df = 2.000, p = 0.744, CFI = 1.000, RMSEA_Estimate = 0.000, [0.000, 0.017], SRMR = 0.001).

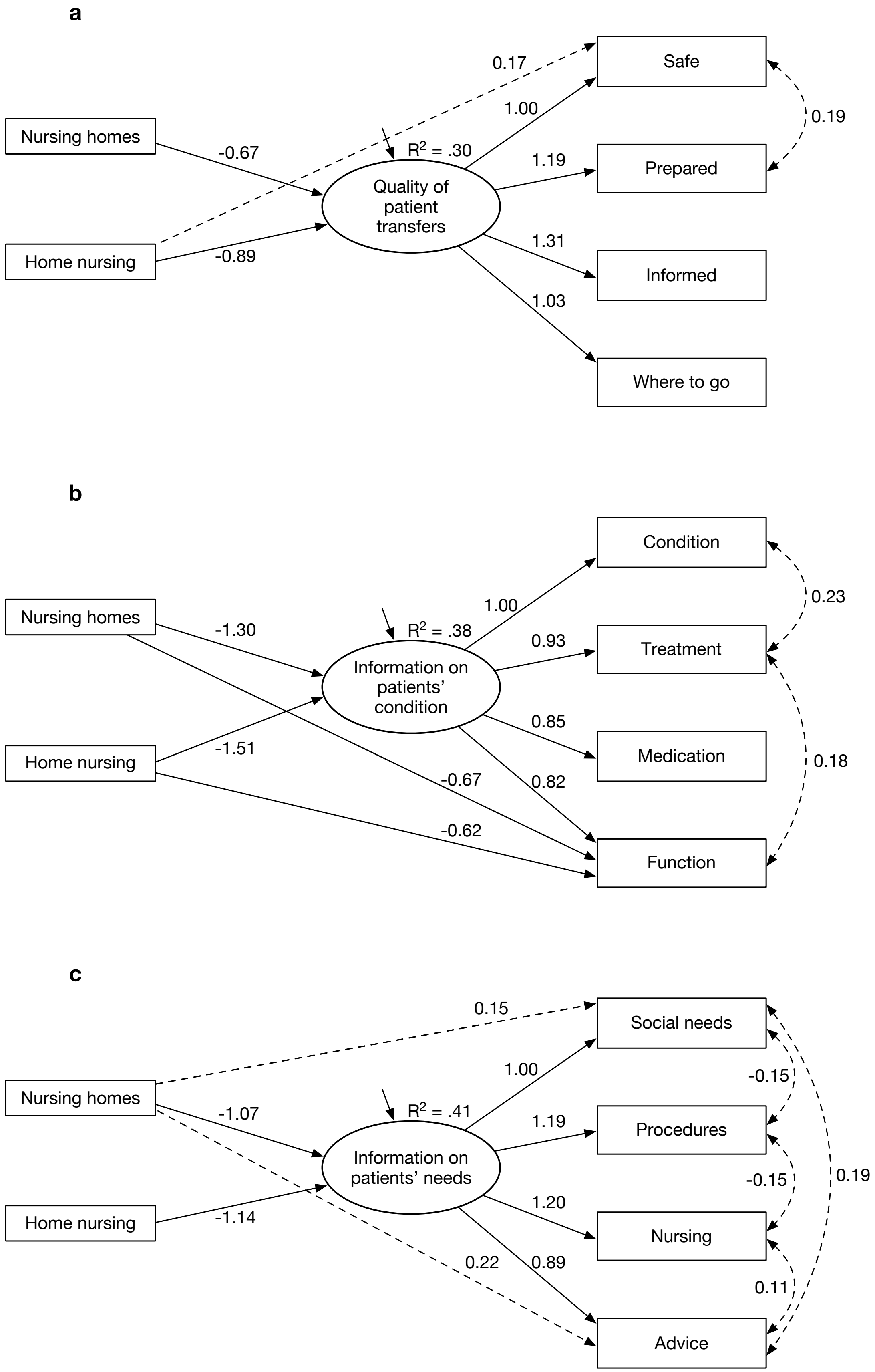

Figure S3: Models with perfect or close to perfect fit, (a) experienced quality of patient transitions, (b) information exchange on patients' condition, and (c) information exchange on patiens' needs. Added parameters (paths) use indicated by broken lines.
